# Libertas: a phase II placebo-controlled study of NRL001 in patients with faecal incontinence showed an unexpected and sustained placebo response

**DOI:** 10.1007/s00384-016-2585-7

**Published:** 2016-04-13

**Authors:** L. Siproudhis, W. Graf, A. Emmanuel, D. Walker, R. Ng Kwet Shing, C. Pediconi, J. Pilot, S. Wexner, J. Scholefield

**Affiliations:** CHU Pontchaillou, Rennes, France; Institution of Surgical Sciences, Akademiska Sjukhuset, Uppsala University, 75185 Uppsala, Sweden; University College Hospital, 235 Euston Road, London, UK; Norgine Ltd, Norgine House, Widewater Place, Moorhall Road, Uxbridge, UB9 6NS UK; Cleveland Clinic Florida, Weston, FL USA; Division of Surgery, University Hospital Nottingham, Nottingham, UK

**Keywords:** Faecal incontinence, Quality of life, Episode frequency, Alpha-1 receptor agonist, NRL001

## Abstract

**Purpose:**

Faecal incontinence (FI) is distressing, significantly reduces quality of life (QoL) and has few pharmacological treatments. The α_1_-adrenoceptor agonist NRL001 (1R,2S-methoxamine hydrochloride) improves anal sphincter tone. NRL001 efficacy was evaluated by changes in Wexner scores at week 4 vs. baseline in NRL001-treated patients compared with placebo. Impact of NRL001 on QoL and safety were also assessed.

**Methods:**

Four hundred sixty-six patients received NRL001 (5, 7.5 or 10 mg) or placebo as suppository, once daily over 8 weeks. Wexner score, Vaizey score and QoL were analysed at baseline, week 4 and week 8. FI episodes and adverse events were recorded in diaries.

**Results:**

At week 4, mean reductions in Wexner scores were −3.0, −2.6, −2.6 and −2.4 for NRL001 5, 7.5, 10 mg and placebo, respectively. All reduced further by week 8. As placebo responses also improved, there was no significant treatment effect at week 4 (*p* = 0.6867) or week 8 (*p* = 0.5005). FI episode frequency improved for all patients, but not significantly compared with placebo (week 4: *p* = 0.2619, week 8: *p* = 0.5278). All patients’ QoL improved, but not significantly for all parameters (*p* > 0.05) except depression/self-perception at week 4 (*p* = 0.0102) and week 8 (*p* = 0.0069), compared with placebo. Most adverse events were mild and judged probably or possibly related to NRL001.

**Conclusions:**

All groups demonstrated improvement in efficacy and QoL compared with baseline. NRL001 was well-tolerated without serious safety concerns. Despite the improvement in all groups, there was no statistically significant treatment effect, underlining the importance of relating results to a placebo arm.

## Introduction

Faecal incontinence (FI) is a distressing condition defined as the inability to voluntarily control the passage of faecal matter or gas through the anal canal and expel it at a socially acceptable time and location [1]. It significantly reduces psychological and emotional well-being and negatively affects quality of life (QoL) [2, 3]. The feelings of embarrassment and depression may be one of the explanations why the majority of patients do not report FI to their physician [4] and may be one of the main reasons why only approximately one third of symptomatic patients seek medical help [5]. Estimates of the prevalence of FI vary depending on the population studied. One survey of adults living in the US community showed that approximately 8.3 % reported FI [6]. The incidence of FI has been shown to be significantly higher in women [7, 8], in patients with existing gastroenterological conditions [8] and also significantly increases with age [9, 10]. The most recent and largest North American study found that almost 20 % of healthy women had experienced FI during the preceding 12 months [9].

Mild FI may be improved with conservative therapies including lifestyle changes and biofeedback retraining. Pharmacological interventions remain poorly investigated, and a recent review of clinical trials suggests that many therapies do not significantly improve symptoms [11]. Surgery is increasingly performed in FI due to the failure of conservative and pharmacological therapies. However, many subjects are poor surgical candidates and long-term success rates are variable [12–14]. The lack of a universally effective, surgical method has led to the development of numerous complex options including an artificial bowel sphincter, magnetic anal sphincter, stimulated graciloplasty and sacral nerve stimulation [15–18]. These procedures might lead to definite improvement, but the morbidity profiles emphasize the need for effective pharmacological treatments.

The internal anal sphincter (IAS) muscle exists in a natural state of tonic contraction. Naturally occurring degeneration due to ageing can result in a loss of smooth muscle tone, and in addition, reduced contractile function might lead to uncontrollable bowel movements [19]. The IAS muscles receive excitatory innervations to mediate contraction or relaxation via the α- or β-adrenergic receptors, respectively. Therefore, the adrenergic sympathetic nervous system could be targeted therapeutically with a view to restoring smooth muscle tone and improving FI symptoms [20].

Topical application of the selective α_1_-adrenoceptor agonist phenylephrine increased anal sphincter tone as measured by mean anal resting pressure (MARP) in both healthy subjects and patients with FI [21, 22]. Symptomatic improvement, however, was disappointing in a separate phenylephrine study [23]. 1R,2S-Methoxamine hydrochloride (NRL001) is a highly selective α_1_-adrenoceptor agonist that is approximately four times more potent than phenylephrine at constricting porcine IAS tissue in vitro [24]. Local administration of NRL001 to healthy subjects and patients with FI has been shown to increase MARP [25–27].

Therefore, the Libertas study—a multi-centre, double-blind, randomized, placebo-controlled, dose-ranging clinical study—was designed to primarily investigate the impact of NRL001 on FI symptoms [28]. Secondary aims were to assess patient QoL, safety profile and tolerability.

## Methods

### Ethics

Independent Ethics Committees approved this study, which was conducted in accordance with Good Clinical Practice and the Declaration of Helsinki, 2008. All patients provided written informed consent prior to enrolment. This study was registered at ClinicalTrials.gov (NCT01656720).

### Study population

Patients aged 18 years or more with a diagnosis of FI (Wexner score of 8–20 [29]) and an intact IAS on ultrasonography were eligible for inclusion. Patients had to have FI for at least 6 months and two or more diary confirmed FI episodes per week in the 4 weeks prior to screening. Patients were excluded if there was evidence of external anal sphincter trauma, which allowed for a more homogenous patient population by reducing variation in anal tone and defects. Patients with uncontrolled gastrointestinal, cardiovascular or obstructive pulmonary diseases were excluded. Those suffering from chronic liver disease, renal impairment or closed-angle glaucoma or other conditions of light sensitivity and/or mydriasis were also excluded. The patient demographics are described in Table 1.

**Table 1:** Summary of patient demographic characteristics (safety population)

Variable		NRL001 5 mg (*N* = 114)	NRL001 7.5 mg (*N* = 115)	NRL001 10 mg (*N* = 122)	Placebo (*N* = 112)	Total (*N* = 463)
Age, years		61.4 (12.37)	62.4 (12.93)	62.9 (12.59)	61.4 (11.39)	62.1 (12.32)
Sex, *n* (%)	Female	98 (86.0)	99 (86.1)	102 (83.6)	91 (81.3)	390 (84.2)
	Male	16 (14.0)	16 (13.9)	20 (16.4)	21(18.8)	73 (15.8)
BMI (kg/m^2^)		26.4 (4.38)	26.6 (4.41)	26.8 (4.99)	27.2 (4.74)	26.8 (4.64)
Height (cm)		164.1 (8.36)	163.2 (7.53)	164.2 (8.37)	165.2 (8.61)	164.1 (8.23)
Weight (kg)		71.2 (13.80)	70.07 (11.82)	72.3 (14.26)	74.2 (14.75)	72.1 (13.73)
Ethnicity, *n* %	White	110 (96.5)	113 (98.3)	116 (95.1)	106 (94.6)	445 (96.1)
	Black or African-American	1 (0.9)	0 (0.0)	0 (0.0)	1 (0.9)	2 (0.4)
	Asian	0 (0.0)	0 (0.0)	1 (0.8)	1 (0.9)	2 (0.4)
	Other	3 (2.6 %)	2 (1.7)	5 (4.1)	4 (1.3	14 (3.0)

Unless noted otherwise, values are expressed as mean (standard deviation)

*n* number of patients, *BMI* body mass index

### Study design

**Fig. 1:**
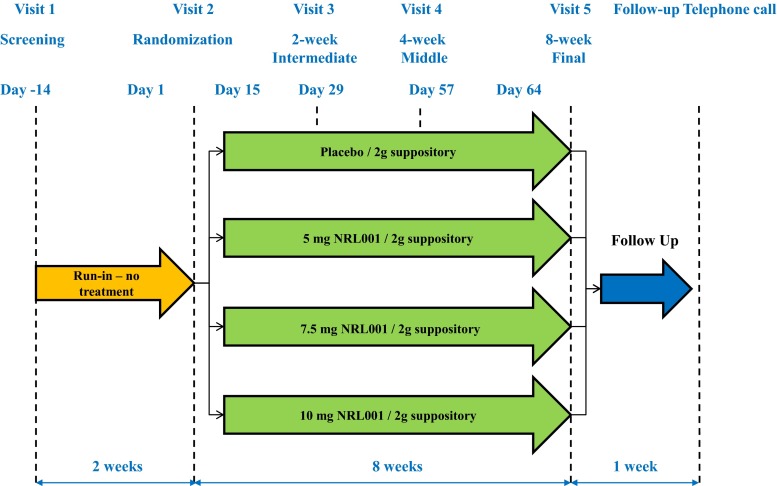
The Libertas study design

In brief, this was a multi-centre, phase II, double-blind, randomized, placebo-controlled, parallel group, dose-ranging study (Fig. 1). Patients were randomized in a 1:1:1:1 ratio as follows: NRL001 5 mg, 7.5 mg, 10 mg or placebo in a 2-g suppository to be self-administered once daily [28]. These doses were based on results of a previous study of NRL001 in healthy volunteers [30].

This study was conducted from 27^th^ of February 2012 to 30^th^ of December 2013, in 55 European centres in the Czech Republic, France, Germany, Hungary, Italy, Poland, Spain, Sweden and the UK.

Patients were assigned a unique number based on the study site number and the serial number of the patient at screening. During randomization, eligible patients were assigned a unique randomization number from the Interactive Web Response System (IWRS; Premier Research Group Ltd, UK) that was linked to two patient investigational medicinal product packs. The investigator maintained a list of patient names, assigned unique patient numbers and associated assigned unique randomization numbers. The password-protected randomization list was supplied by a statistician of Premier Research Group Ltd using the Statistical Analysis System (SAS) for Windows (SAS Institute Inc., USA) to Pharmaceutical Development, Norgine Ltd, UK. NRL001 and placebo were packed and labelled according to pertinent regulations by the Sponsor. The randomization schedule was then made available to the Premier Research Group Ltd IWRS.

This was a double-blind study with all NRL001 and placebo suppositories provided in the same packaging and labelling. No person involved in conducting the study had access to the randomization code before the blinding was officially broken. However, in the case of an emergency that required the investigator to be unblinded, the investigator was allowed to obtain the randomization code via the IWRS.

The study period consisted of a 2-week run-in phase, an 8-week treatment period and a 1-week post-treatment follow-up. In total, the study comprised five visits to the study site. Patients were screened on day 14. On day 1 (randomization), patients received their first dose, provided the eligibility criteria were met. Patients attended visits at week 2, week 4 and week 8. A follow-up telephone call was made at week 9 to assess overall patient satisfaction and the duration of any adverse event.

Patients received 70 suppositories in total—35 at week 1 and 35 at week 4—and were provided with an electronic diary (e-Diary) to record details of suppository administration and frequency and type of incontinence episodes.

The primary endpoint of this study was to determine the efficacy of NRL001 as assessed by a change in the Wexner score at week 4 in patients receiving NRL001 compared with those receiving placebo. Key secondary endpoints included Vaizey scores and the number of FI episodes per week as additional efficacy parameters at week 4 and week 8, as well as patient QoL and satisfaction.

### Sample collection and analysis

The Wexner scoring system consisted of the score sum of five parameters (frequency of gas, liquid or solid incontinence, need to wear a pad and lifestyle alterations) scored on a scale of 0 (absent) to 4 (daily) [29]. A total score of 0 suggested full continence and score of 20 complete FI. The Vaizey scoring system was subsequently created by modifying to include two additional questions assessing the ability to defer defecation and use of antidiarrheal medication [31]; 0 suggested full continence and 24 complete FI.

QoL was measured using the FI quality of life (FIQoL) scale [2] and the EQ-5D-5L Healthcare Questionnaire [32, 33] at baseline, week 4 and week 8. The FIQoL scale ranged from 1 to 5, where 1 indicated lowest QoL and 5 the highest, and was produced by scoring statements based on lifestyle, coping/behaviour, depression/self-perception and embarrassment. The EQ-5D-5L Healthcare Questionnaire assessed five parameters: mobility, self-care, ability to perform usual activities, pain/discomfort and anxiety/depression. It also included a visual analogue scale (VAS) for patients to record their self-rated health state. The frequency of FI episodes was assessed using recordings made in the e-Diaries.

Pharmacokinetic relationships between NRL001 concentration and either efficacy or adverse effects were assessed at week 1 (pre-dose and 1, 2 and 4 h post-dose) and week 4.

### Safety assessment

Adverse events were recorded from screening until the end of the study and were coded using MedDRA Version 14.0 or higher. All adverse events were graded as mild, moderate or severe according to intensity, and the relationship to NRL001 was classified as probable, possible or unrelated. The final phone call was used to assess the patients’ global perception of efficacy and the duration of adverse events.

Blood pressure, pulse rate, physical examination, urine analysis and 12-lead electrocardiogram measurements were recorded at each visit. Twenty-four-hour Holter monitoring was performed at screening and week 2. Patients also had blood samples taken at screening, week 2 and week 8 for laboratory safety tests of clinical chemistry and haematology.

### Statistical analysis

The sample size was calculated using a responder rate. Active treatment was classed as a success if it showed a 20 % improvement over placebo in the primary endpoint. With 80 % power and a two-sided level of significance of 0.05 (5 %), a minimum of 98 patients were required in each treatment group to show a difference between rates of 25 and 45 %.

The primary endpoint was defined as the change relative to baseline in Wexner score at week 4. Variation amongst the four treatment groups was tested using an analysis of covariance (ANCOVA) with screening as covariate, and statistical significance was defined as *p* < 0.05. Differences between placebo and each of the three active treatment groups are presented with a 95 % confidence interval (CI). Where significant variation was seen, pairwise differences between placebo and each of the three active treatment groups were tested by calculating CIs for the difference in least square means between placebo and active treatment using Dunnett’s method within the ANCOVA. Differences in the number of FI episodes and the Vaizey, and FIQoL scores were tested using the same statistical methods as the primary endpoint. Changes in the EQ-5D-5L questionnaire from screening to week 4 and week 8, and the overall assessment of patient satisfaction at week 9 were tested using the Wilcoxon test.

Plasma concentrations of NRL001 were summarized for each treatment group, and the relationship between estimated areas under the concentration-time curve (AUC) determined.

Study populations included the safety population, all patients who received any dose of NRL001 or placebo; the modified intent to treat (mITT) population, which was used to analyse efficacy and QoL, and included all patients who received any dose of NRL001 or placebo and had data on the Wexner score at both screening and week 4; and the pharmacokinetic population, all patients who received any dose of NRL001 and had valid pharmacokinetic data.

## Results

### Patients

**Fig. 2:**
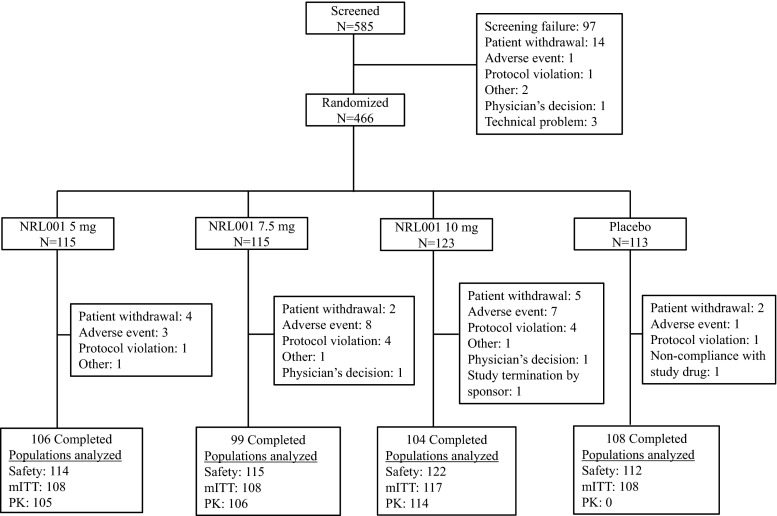
Consort patient flow diagram

Approximately 580 patients were planned to be screened to obtain 400 evaluable patients. A total of 466 patients were randomized and 463 (safety population) received at least one dose of NRL001 (5 mg, *n* = 114; 7.5 mg, *n* = 115; 10 mg, *n* = 122) or placebo (*n* = 112, Fig. 2). The mean age was 62.1 years and the majority were female (390/463 [84 %], Table 1). The mITT population comprised 440 patients (NRL001 5 mg, *n* = 108; 7.5 mg, *n* = 108; 10 mg, *n* = 117 and placebo *n* = 107). A total of 23 patients from the safety population were excluded from the mITT population (15 because of missing data on the Wexner score at baseline and/or week 4, and a further eight patients were excluded as a consequence of GCP deviations at one particular study centre). The pharmacokinetic population comprised 325 patients (NRL001 5 mg, *n* = 105; 7.5 mg, *n* = 106; and 10 mg, *n* = 114).

### Efficacy

**Fig. 3:**
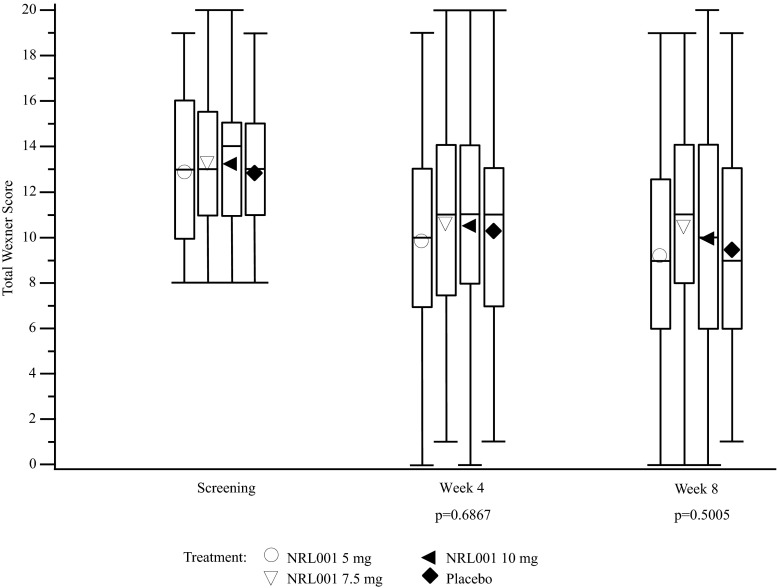
Patients’ Wexner scores at screening, week 4 and week 8 (mITT population). *Symbols* are the means, *boxes* are the interquartile range, *lines within the boxes* are the median and *whiskers* are the range. No significant treatment effect of the change from screening was observed at week 4 or week 8 (*p* values calculated using ANCOVA with screening as covariate)

At baseline, mean Wexner scores were similar between the NRL001 and placebo groups (12.9 ± 3.1 for NRL001 5 mg, 13.3 ± 3.2 for NRL001 7.5 mg, 13.3 ± 3.1 for NRL001 10 mg and 12.9 ± 3.0 for placebo, Table 2). No statistically significant treatment effect was detected at week 4 or week 8 (Fig. 3).

**Table 2:** Mean (SD) screening and mean (SD) (95 % CI) changes from screening of Wexner and Vaizey scores, and the number of faecal incontinence episodes per week at week 4 and week 8 following treatment with NRL001 or placebo (mITT population)

Parameter	NRL001 5 mg		NRL001 7.5 mg		NRL001 10 mg		Placebo		Effect of treatment	
									(*p* value)^a^	
	Week 4	Week 8	Week 4	Week 8	Week 4	Week 8	Week 4	Week 8	Week 4	Week 8
Wexner scores										
Screening	12.9 (3.11) (*n* = 108)		13.3 (3.21) (*n* = 108)		13.3 (3.07) (*n* = 117)		12.9 (3.00) (*n* = 107)			
Change	−3.0 (4.04) (*n* = 108)	−3.6 (4.73) (*n* = 104)	−2.6 (3.53) (*n* = 108)	−3.1 (3.80) (*n* = 94)	−2.6 (3.73) (*n* = 117)	−3.3 (4.41) (*n* = 105)	−2.4 (3.97) (*n* = 107)	−3.5 (4.46) (*n* = 106)	0.6867	0.5005
95 % CI	[−1.75–0.63]	[−1.53–1.21]	[−1.41–0.97]	[−1.00–1.81]	[−1.37–0.96]	[−1.15–1.58]				
Vaizey scores										
Screening	15.4 (3.70) (*n* = 108)		15.5 (3.57) (*n* = 108)		15.3 (3.62) (*n* = 116)		15.3 (3.52) (*n* = 107)			
Change	−3.3 (4.82) (*n* = 108)	−4.5 (5.53) (*n* = 104)	−3.0 (4.43) (*n* = 108)	−4.1 (4.77) (*n* = 93)	−3.0 (4.56) (*n* = 115)	−3.8 (5.14) (*n* = 103)	−3.1 (4.61) (*n* = 105)	−4.0 (5.26) (*n* = 105)	0.9429	0.7794
95 % CI	[−1.62–1.23]	[−2.06–1.21]	[−1.32–1.53]	[−1.72–1.65]	[−1.31–1.50]	[−1.39–1.89]				
No. of FI episodes										
Screening	16.37 (17.16) (*n* = 105)		17.03 (17.79) (*n* = 106)		17.38 (20.17) (*n* = 116)		19.31 (21.97) (*n* = 104)			
Change	−3.4 (14.41) (*n* = 102)	−6.2 (11.64) (*n* = 93)	−3.3 (8.58) (*n* = 91)	−5.0 (10.70) (*n* = 86)	−5.2 (9.86) (*n* = 108)	−7.0 (10.48) (*n* = 96)	−6.3 (14.65) (*n* = 102)	−7.4 (15.55) (*n* = 94)	0.2619	0.5278
95 % CI	[−0.24–7.30]	[−1.68–6.52]	[−0.79–6.99]	[−1.71–6.71]	[−2.50–4.93]	[−3.88–4.31]				

^a^ *p* values calculated using ANCOVA with screening as covariate. No significant treatment effect was observed between groups

*SD* standard deviation, *CI* confidence interval, *mITT* modified intent to treat, *n* number of patients, *FI* faecal incontinence

Vaizey scores and the number of FI episodes per week decreased from screening to week 4 for all treatment groups and had decreased further by week 8. However, no statistically significant treatment effect was detected (Table 2). Responses in the placebo-treated group were similar to those receiving NRL001 and persisted through the 8-week study period.

### Quality of life

**Fig. 4:**
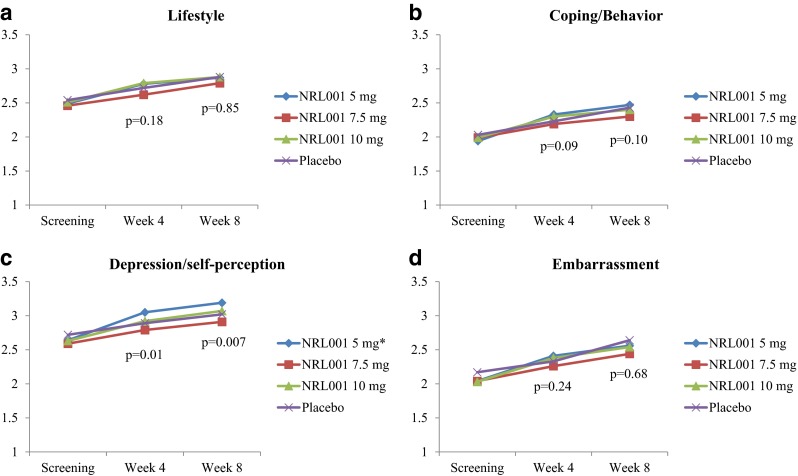
Patients’ mean FIQoL scores at screening, week 4 and week 8 (mITT population) for the four parameters: lifestyle (**a**), coping/behaviour (**b**), depression/self-perception (**c**) and embarrassment (**d**). *p* values for treatment effects calculated using ANCOVA with screening as covariate. *Pairwise differences between placebo and NRL001 5 mg were statistically significant at both week 4 and week 8 at the 95 % CI, calculated using Dunnett’s method within ANCOVA

At baseline, mean FIQoL scores were similar for NRL001 and placebo groups in all four parameters (Fig. 4). Scores for each parameter increased in all treatment arms at week 4 and increased further by week 8. Statistically significant improvements of NRL001 treatment effects compared with placebo were observed on depression/self-perception at week 4 (*p* = 0.0102) and week 8 (*p* = 0.0069) but not for the other scales (*p* > 0.05). Analysis of 95 % CIs revealed a statistically significant treatment difference between NRL001 5 mg and placebo for depression/self-perception at both week 4 (treatment difference 0.25 [95 % CI 0.06–0.44]) and week 8 (treatment difference 0.28 [95 % CI 0.08–0.47]) but not for other doses.

There was a significant increase in overall assessment of health according to the VAS at week 4 in all NRL001 treatment groups compared with placebo (*p* = 0.0478), but VAS was not significantly improved at week 8 in any of the treatment groups compared with placebo (*p* > 0.05, data not shown). Analysis of EQ-5D-5L data showed marginal differences compared with placebo at week 4 and week 8 for some of the parameters (Table 3).

**Table 3:** Wilcoxon *p* values calculated for the change in the EQ-5D-5L Healthcare Questionnaire from screening at week 4 and week 8 (mITT population)

Question	Comparison of NRL001 vs. placebo	Wilcoxon *p* value	
		Week 4	Week 8
Mobility	5 mg	0.00353	0.34432
	7.5 mg	0.16309	0.68848
	10 mg	0.05261	0.78593
Self-care	5 mg	0.12285	0.32080
	7.5 mg	0.07852	0.49652
	10 mg	0.44415	0.61766
Activity	5 mg	0.0007561	0.03675
	7.5 mg	0.06087	0.02401
	10 mg	0.02297	0.13918
Pain	5 mg	0.15276	0.28540
	7.5 mg	0.33317	0.57655
	10 mg	0.14695	0.38680
Anxiety/depression	5 mg	0.18180	0.07648
	7.5 mg	0.73124	0.68457
	10 mg	0.34466	0.94216

*mITT* modified intent to treat

### Pharmacokinetics

There was a dose-dependent increase in the plasma concentration of NRL001 with mean AUC values of 15.4 ± 8.7 ng/mL/h for NRL001 5 mg, 23.9 ± 12.9 ng/mL/h for NRL001 7.5 mg and 32.2 ± 17.0 ng/mL for NRL001 10 mg (pharmacokinetic population). No NRL001 was detected in the placebo-treated group (data not shown).

### Patient satisfaction

Seventy-one (82.6 %), 59 (74.7 %) and 72 (75.8 %) patients who received NRL001 5, 7.5 and 10 mg, respectively, and 77 (85.6 %) patients who received placebo said they would choose to take the same medication again. There were no statistically significant differences between NRL001 groups and placebo (*p* > 0.05) for overall assessment of patient satisfaction (performance of study medication; taking study medication again and the change in QoL).

### Safety

Overall, 212/463 (46 %) patients reported 494 adverse events (Table 4). In general, more patients in the NRL001-treated groups were affected by adverse events (45.2–52.5 %) than those who received placebo (36.6 %). The most frequent adverse events following dosing with NRL001 (paraesthesia, feeling cold and piloerection) were expected, generally mild in intensity and judged to be attributed to the pharmacological effect of NRL001, but the relationship between adverse events and efficacy of NRL001 was not explored.

**Table 4:** Adverse events reported by ≥3 % of patients (safety population)

	NRL001 5 mg (*N* = 114)		NRL001 7.5 mg (*N* = 115)		NRL001 10 mg (*N* = 122)		Placebo (*N* = 112)		Total (*N* = 463)	
	*n* (%)	*e*	*n* (%)	*e*	*n* (%)	*e*	*n* (%)	*e*	*n* (%)	*e*
Any event	55 (48.2)	107	52 (45.2)	140	64 (52.5)	162	41 (36.6)	85	212 (45.8)	494
Paraesthesia	9 (7.9)	9	14 (12.2)	18	17(13.9)	21	1 (0.9)	1	41 (8.9)	49
Headache	1 (0.9)	1	6 (5.2)	7	5 (4.1)	5	6 (5.4)	6	18 (3.9)	19
Feeling cold	11 (9.6)	11	7 (6.1)	7	12 (9.8)	13	3 (2.7)	4	33 (7.1)	35
Chills	8 (7.0)	8	6 (5.2)	8	8 (6.6)	10	0 (0.0)	0	22 (4.8)	26
Piloerection	5 (4.4)	7	8 (7.0)	9	14 (11.5)	15	0 (0.0)	0	27 (5.8)	31
Urinary tract infection	4 (3.5)	7	1 (0.9)	1	5 (4.1)	5	4 (3.6)	4	14 (3.0)	17
Other	17 (14.9)	64	10 (8.7)	90	3 (2.5)	93	27 (24.1)	70	57 (12.3)	317

Includes events occurring in less than 3 % of patients

*n* number of patients, *e* number of events

Eighteen patients (3.9 %) experienced adverse events leading to withdrawal from the study, and 20 patients (4.3 %) experienced adverse events that led to discontinuation of the study drug. The number of patients whose adverse events led to discontinuation of the study drug was higher in the NRL001-treated groups than placebo (19/20 receiving NRL001; 1/20 receiving placebo). One patient who received NRL001 10 mg developed severe cardiac failure following hospitalization from urosepsis that was considered serious and judged possibly related to study drug, although this resolved by the end of the study. There were no reports of bradycardia during the study.

## Discussion

Wexner scores decreased across all NRL001 treatment arms compared with baseline. However, a marked placebo response was observed during the entire 8-week treatment period such that no statistically significant treatment effect was observed when comparing active treatment with placebo. Secondary endpoints showed similar findings, with marked improvements in response to NRL001 accompanied by a notable placebo response of similar magnitude. However, NRL001 had a statistically significant effect on some QoL assessments at week 4 and week 8 compared with placebo: NRL001 significantly improved patients’ self-assessed mobility, activity and overall health at week 4 and activity at week 8. Although these were not paired with statistically significant improvements in Wexner scores, correlations were apparent. Decreases in Wexner and Vaizey scores, and frequency of FI episodes complemented QoL improvements; associations have proven to be significant in previous validation studies [34–36].

The reduction in Wexner score after 8 weeks of treatment when compared with baseline was highly significant for all NRL001 treatment arms (*p* < 0.0001) as well as placebo. Pharmacokinetic data demonstrated a dose-dependent increase in plasma concentrations of NRL001. Visual inspections of the data indicated that there was no dose-proportional improvement in any of the endpoints, although this was not tested statistically. A dose-dependent increase in MARP was reported in a study in which NRL001 was administered to healthy volunteers [27]. Assessment of anorectal physiology may have provided useful information regarding the mechanistic insight of the drug. However, anorectal physiology assessments were likely to have significantly adversely affected enrolment feasibility and cost. Furthermore, evaluations of anorectal physiology, such as anorectal manometry assessments, are often also poorly correlated with patient outcomes [37, 38]. This has been hypothesized to be due to a stenosing effect following surgery, increasing the resistance to blood flow through the anus, masking physiological alterations detected in pressure measurements [39]. Additionally, MARP was not assessed in this current study due to the difficulties standardizing methodologies in a large patient population and the suggestion that many therapies do not significantly improve symptoms of FI, despite their positive effect on MARP [11]. This and the lack of dose-response in symptom improvement in the current study argue strongly that biological endpoints such as MARP are poor markers of patient outcomes. The FI episodes reported in this study encompassed all forms of incontinence. It was considered that analysis of faecal matter incontinence episodes (FMIE), which excludes flatus, might reveal a more robust treatment effect. Consequently, although not presented here, FMIE for each active treatment group at both week 4 and week 8 were analysed and compared with those of placebo-treated patients but no significant treatment effect was observed. As such, evaluation of Wexner scores as the primary endpoint for this study was considered appropriate.

The reported adverse events were similar to those in previous studies (paraesthesia, chills and piloerection) and were thought to be a direct result of increased α_1_-adrenoceptor stimulation [25–27, 30, 40, 41]. Visual inspections of the data indicated that adverse events were less frequent in the placebo-treated group than any of the NRL001-treated groups. There also appeared to be no dose-relationship in the frequency of adverse events reported across the three active treatment groups, although not tested statistically. Safety assessments did not show any new or previously unknown risks of NRL001. Bradycardia—an expected effect of α_1_-adrenoceptor stimulation [42]—was not reported in any treatment group. A meta-analysis of previous studies involving topical application of NRL001 has shown that whilst patients generally experienced a dose-dependent decrease in heart rate, bradycardia was not clinically significant [43]. Therefore, the safety of NRL001 was deemed better than expected.

The inclusion criteria for this study included a screening Wexner score of 8–20. Therefore, patient FI severities ranged from relatively mild to very severe [29]. It is possible that the severity of Wexner score at screening was an important factor; however, a descriptive post hoc analysis (Norgine data on file) was unable to show any difference in effect between patients with baseline Wexner score of 8–11, 12–15 or 16–20. This inclusion criterion was designed to make the results of this study comparable with those of other recent studies that assess new therapeutic options for treatment of FI (not direct surgical repair) and use the Wexner score to define their patient population [44–46]. Prior exposure of patients to biofeedback therapy, a conservative approach employed to treat FI based on the theories of operant conditioning, was not taken into consideration. Patients receiving conservative treatments display sustained FI symptom improvement for up to a year, but some studies hypothesize that beneficial effects relate more to the relationship with the therapist than the technical aspects of the therapy and many responders lose the effect over time [47]. It is possible that any patients naive to biofeedback therapy included in this study may have benefited more from inclusion in the study rather than the pharmacological substance itself. Also, any patient in whom biofeedback therapy had previously improved symptoms, but which was lost over time, might be more likely to have improvement in their symptoms again. As such, this study may have benefited by including a run-in period with biofeedback therapy to exclude those patients who would have attained adequate FI symptom relief from conservative treatments. However, this is likely to have restricted the number of patients eligible for inclusion.

A number of important factors made the Libertas study unique: There have been few similarly designed studies in FI involving this number of patients; this study was the first testing NRL001 in a broad population of patients; and it employed innovative strategies, including the use of an outreach program, to recruit patients whilst taking into account the sensitivity of the condition [28], and finally the comparison of subjects’ responses to NRL001 with placebo, rather than solely with screening measurements. Given the positive effects of NRL001 in previous studies [25–27], the findings of the current study are highly relevant, confirming the importance of a placebo group.

Predictors of the placebo response have been a focus of many studies with potentially significant impact on clinical trials. The placebo response observed in this study was larger than expected and did not decline during the 8-week treatment period. The placebo suppositories used in this study comprised 0.03-g colloidal anhydrous silica and 1.97-g hard fat. NRL001 suppositories contained reduced amounts of fat to compensate for the addition of NRL001. The route of administration of a treatment is known to impact the extent to which a placebo response may be elicited. A review of placebo responses observed in clinical trials of migraine treatments concluded that interventions administered at the site of pain elicit a greater placebo response than those administered at a distance [48]. As such, the placebo response observed in this study is likely to have been more robust than a placebo response observed had the intervention for FI been administered orally. Furthermore, progressively invasive treatments are coupled with a more robust placebo response: One study of a migraine treatment reported pain relief of 39 % following administration of placebo as a suppository, whereas others have demonstrated pain relief of 39–32 % following administration via nasal sprays [48]. Placebo-controlled studies in patients with FI have also shown unexpected high response rates after saline injection [49] or sham electrical stimulations [50, 51]. As well as the route of administration, the small sample sizes of these studies may have contributed to the lack of clinical effect in these studies. This was one factor that the Libertas study aimed to mitigate, and by using a broad and ample study population, it was hoped that the sample size would be sufficient to demonstrate a significant clinical effect. These findings suggest that FI studies that report a positive treatment effect but do not make comparisons with placebo should perhaps be interpreted with caution.

Treatment-specific restrictions on conservative therapies such as dietary advice, behavioural changes or muscle-strengthening exercises were not imposed on patients during this study. Therefore, active treatment groups and the placebo-treated group should have received similar treatments throughout. Although conservative medical treatments were not analysed here, a Cochrane review of 21 individual studies found little evidence to support the therapeutic benefits of anal sphincter exercises or biofeedback therapy alone [52], and so the use of these by placebo-treated patients would have been unlikely to account for their robust response. Patients were also required to report any concomitant medications taken throughout the study in their e-Dairies. A total of 17 patients used drugs for the treatment of functional gastrointestinal disorders during the course of the study, although there was imbalance in relation to treatment group. Therefore, the use of concomitant medications or conservative medical treatments was not thought to differ between treatment groups and thus contribute to the response observed in the placebo-treated group.

A recent review suggested that the placebo response is also related to cognitive constructs, such as locus of control—the extent to which individuals believe they can control events [53, 54]. In particular, a placebo response is seen in those with an external locus of control, meaning participants have a strong belief that outcomes are determined by factors external to their control [55, 56]. Additional factors that appear to minimize the placebo response in gastroenterology studies are the use of a randomized, double-blind, controlled, parallel group study design, with dosing taking place no more than once daily [57, 58], all of which were implemented in this study.

In conclusion, patients in this study displayed an improvement in FI symptoms in all parameters tested throughout the duration of this 8-week study. This was also reflected in improvements of QoL and positive patient satisfaction. However, no statistically significant treatment effects of NRL001 were seen compared with placebo because of the comparable response observed in this group. This finding confirms the importance of robust study design in clinical trials to include appropriate control and comparator groups and should be considered when interpreting other studies in this therapeutic area. Libertas was intended to provide a framework for future studies allowing clear endpoints to be derived. It is hoped that, despite the lack of a treatment effect in the Libertas study, lessons learned from its design and conduct will ultimately benefit patients suffering with FI.
